# Use of the CONSIDER statement by eye health researchers when conducting and reporting research involving Indigenous peoples: an online survey

**DOI:** 10.1038/s41433-023-02881-6

**Published:** 2024-01-09

**Authors:** Isaac Samuels, Lisa M. Hamm, Juan Carlos Silva, Benoit Tousignant, João M. Furtado, Lucy Goodman, Renata Watene, Jaki Adams, Indigenous Eye Health Research Consortium, Aryati Yashadhana, Ben Wilkinson, Helen Dimaras, Ilena Brea, Jaymie Rogers, Joanna Black, Joshua Foreman, Juan Camilo Arboleda, Juan Francisco Yee, Julián Trujillo, Lisa Keay, Luisa Casas Luque, María del Pilar Oviedo-Cáceres, Martha Saboya, Monica Alves, Myrna Lichter, Pushkar Silwal, Rebecca Findlay, Rosario Barrenechea, Samantha Simkin, Sharon Bentley, Shelley Hopkins, Solange Rios Salomão, Stuti Misra, Tim Fricke, Tulio Reis, Jacqueline Ramke, Matire Harwood

**Affiliations:** 1 Department of Ophthalmology, University of Auckland, Auckland, 1142 New Zealand; 2 School of Optometry and Vision Science, University of Auckland, Auckland, 1142 New Zealand; 3 Independent Researcher, Bogota, 110111 Colombia; 4 School of Optometry, Université de Montréal, Montreal, H3T 1P1 QC Canada; 5 Department of Social and Preventive Medicine, School of Public Health, Université de Montréal, Montreal, H3C 3T4 QC Canada; 6 Division of Ophthalmology, Ribeirão Preto Medical School, University of São Paulo, Ribeirão Preto, 14049900 Brazil; 7 The Fred Hollows Foundation, Darwin, Australia; 8 International Centre for Eye Health, London School of Hygiene and Tropical Medicine, London, WC1E 7HT UK; 9 School of Population Health, University of Auckland, Auckland, 1142 New Zealand; 10 University of New South Wales, Sydney, NSW Australia; 11 Capital and Coast District Health Board, Wellington, New Zealand; 12 The Hospital for Sick Children, Toronto, Canada; 13 Pan American Health Organization, Panama, Panama; 14 Centre for Eye Research Australia, Melbourne, VIC Australia; 15 Pan American Health Organisation, Bogotá, Colombia; 16 Visualiza, Guatemala, Guatemala; 17 Ministry of Health and Social Protection, Bogotá, Colombia; 18 The International Agency for the Prevention of Blindness, Bogotá, Colombia; 19 University of Santo Tomas, Bucaramanga, Colombia; 20 Pan American Health Organization, Washington, DC USA; 21 University of Campinas, Campinas, Brazil; 22 University of Toronto, Toronto, Canada; 23 National Ministry of Health Directorate and Research, Buenos Aires, Argentina; 24 Queensland University of Technology, Kelvin Grove, QLD Australia

**Keywords:** Epidemiology, Scientific community

## Abstract

**Background:**

Indigenous peoples experience worse eye health compared to non-Indigenous peoples. Service providers and researchers must avoid perpetuating this inequity. To help achieve this, researchers can use the *CONSolIDated critERia for strengthening the reporting of health research involving Indigenous peoples* (CONSIDER) statement. This study aimed to identify the degree to which the CONSIDER statement has been used by eye health researchers when conducting and reporting research with an Indigenous component, and how they perceive its relevance in their future research.

**Methods:**

We used purposive sampling to recruit eye health researchers from any country who have undertaken research with an Indigenous component. The online survey collected quantitative and qualitative data and was analysed using descriptive statistics and reflexive thematic analysis. Responses were gathered on a four-point Likert scale (1 to 4), with four being the most positive statement.

**Results:**

Thirty-nine eye health researchers from nine countries completed the survey (Aotearoa New Zealand, Argentina, Australia, Brazil, Canada, Colombia, Guatemala, Panama, Peru); almost two-thirds (*n* = 24) undertake epidemiological research. On average, participants disclosed only ‘sometimes’ previously reporting CONSIDER items (2.26 ± 1.14), but they thought the items were relevant to eye health research and were motivated to use these guidelines in their future research. Some participants requested clarity about how CONSIDER aligned with existing guidelines, and when and how to apply the statement. Others shared rich experiences of the benefits to their research of Indigenous leadership and collaboration.

**Conclusions:**

The CONSIDER statement is perceived as a valuable tool by these eye health researchers, and there are opportunities to maximise uptake and use, including increasing awareness of the statement, clarity about when it applies, and availability of institutional-level support.

## Introduction

The last few decades have seen significant gains in health outcomes globally, but not equitably for all [1]. Numerous national and international governing bodies have highlighted the health inequity experienced by Indigenous peoples. As a result of colonisation and corresponding loss of land, culture and language, as well as persistent structural discrimination, there are differences in access to, and quality of care at, health services [2–4]. Unfortunately, these inequities are also present—but generally under-researched—within eye health, and exacerbated by a lack of cultural safety within the system [5–7]. Inequity in eye health has been most extensively explored for Indigenous peoples in Australia (Aboriginal and Torres Strait Islander Peoples), and to a lesser extent for Māori peoples in Aotearoa New Zealand and Indigenous communities within North and South America and elsewhere [8–12]. Indeed, the 2021 *Lancet Global Health* Commission on Global Eye Health considered Indigenous populations as one of the groups most underserved by eye health services [12].

In some settings, health research has unfortunately played a role in perpetuating inequities [13]. In response, several guidelines have been developed to protect Indigenous communities and promote culturally safe [5], collaborative research practice [14–18]. Most of these are country specific [14, 15, 17, 18], however attempts have been made to pool research guidelines across countries [16]. In an attempt to synthesise existing guidelines for consistent global reporting, in 2019 collaborators from Australia and Aotearoa developed the *CONSolIDated critERia for strengthening the reporting of health research involving Indigenous peoples* (CONSIDER) statement [19]. The CONSIDER statement is a tool for researchers to enhance the design and reporting of their research, such as honouring Indigenous principles and engaging Indigenous communities, governing bodies and researchers. The statement includes eight domains (governance, prioritisation, relationships, methodologies, participation, capacity, analysis and interpretation and dissemination) covering 17 checklist items (listed in supplementary table 1) relating to health research with an Indigenous component (defined in Box 1) [19].

International calls for reconciliation and Indigenous self-determination in health research, and the growing acknowledgement of inequity in eye health strengthen the rationale to investigate the role of the CONSIDER statement within eye health research [20]. Since CONSIDER is a relatively new tool, we wished to draw eye health researcher’s attention to it, and to establish a baseline of the current practice of active researchers. This study aimed to identify the degree to which the CONSIDER statement has been used previously by eye health researchers, and to understand its perceived relevance going forward.

Box 1 What is research with an ‘Indigenous component’? As outlined by the authors of the CONSIDER statement: [19]

1. Research in which Indigenous identity is a criterion for participation
2. Research in which identity or membership of an Indigenous community is used as a variable for data analysis in which interpretation of data refers directly to Indigenous peoples
3. Research that seeks Indigenous knowledge
4. Research that is likely to affect the health of Indigenous Peoples
5. Research conducted on Indigenous lands

## Methods

The online survey was conducted online between 28 July and 1 November 2022 using Qualtrics software (Qualtrics, 2022; Utah, USA). Ethical approval was obtained from the University of Auckland Human Participants Ethics Committee (Ref: UAHPEC24369) and all participants provided informed consent. The study is reported according to the relevant items of the CHERRIES and CONSIDER guidelines (supplementary tables 2 and 3) [19, 21]. Participants who completed the survey were invited to join the manuscript authorship group; those who agreed were sent to a separate form on completion of their survey. Thus, names of participants and their responses were recorded separately and responses were anonymous. Only two authors (LH, JR) had access to the data. The IP address of each computer submitting a response was checked for duplicate submissions. No reimbursement was offered to participants.

### Participants

Purposive sampling was used whereby members of the research team disseminated an invitation via email on two separate occasions to individuals and organisations within their networks (e.g. Departments of Optometry or Ophthalmology, Schools of Public Health, the Indigenous Peoples Special Interest Group of the International Agency for the Prevention of Blindness) thought to have experience in eye health research with an Indigenous component. When contacting an organization, we requested that our invitation be shared with all relevant researchers in the Department. We also invited authors of studies involving Indigenous populations identified from an ongoing methodological review of eye health surveys that members of the research team are conducting [22]. People from any country were eligible to participate if they self-reported having conducted eye health research with an Indigenous component (Box 1) that resulted in a written report in the peer-reviewed literature or provided to decision-makers. We did not establish a target sample size, but rather attempted to disseminate the invitation as broadly as possible in regions across the globe.

### Research Team

The research team included senior (MH) and emerging (IS, RW, JA) Indigenous researchers who were involved in all phases of the project. Other team members have extensive experience implementing research projects with an Indigenous component.

### Data collection

The questionnaire was developed by one author (LH) and refined after other authors tested its usability and technical functionality. After providing informed consent, participants answered a set of introductory questions. These included whether the participant had heard of the CONSIDER statement prior to the survey, the country in which they conducted most of their eye health research, their field of research, as well as whether they identify as Indigenous. We then asked participants to reflect on whether their eye health research had an Indigenous component (Box 1). If the participant did not think their research met any of the criteria they were diverted to the end of the survey.

Participants reporting an Indigenous component to their research were presented with each of the 17 CONSIDER items in turn, with the order of presentation randomised at the domain level i.e. participants randomly began with the ‘Governance’ items, others with the ‘Relationships’ items and so on. Each item was presented individually, with the definition and examples of the item available by hovering over a prompt. Each item was explored with three compulsory questions (and corresponding 4-point Likert scale, each scored from 1 to 4 in the following order):

1. How often have you included this in your previous reports of eye health research with an Indigenous component? (never/sometimes/most of the time/always);
2. To what extent do you agree or disagree that reports of good eye health research with an Indigenous component should include this item? (strongly disagree/somewhat disagree/somewhat agree/strongly disagree); and
3. How likely are you to include this in your future reports of eye health research with an Indigenous component? (extremely unlikely/somewhat unlikely/somewhat likely/extremely likely).

If a participant reported being extremely or somewhat unlikely to report the item in the future (question 3), two additional questions were posed which were optional: ‘What is the main concern that would prevent you from describing this in your future reports of health research involving Indigenous peoples?’ and ‘Is there anything that would help you to report this item in future?’. If a participant indicated something could enable future reporting, they were prompted to describe this in a free text box. After each item there was an option to leave a free text comment about the item or response. Each item was presented on a new page/screen of the survey. A back button allowed participants to review their responses prior to submission.

### Analysis

We used descriptive statistics (mean and standard error of the mean) to summarise outcomes. Qualitative responses for each item were collated and analysed by two researchers independently (LH, IS). Inductive coding was initiated after familiarisation [23, 24]. Themes were developed iteratively by discussing the meaning of participant responses with similar codes, updating codes and potential themes in light of the thoughtful discussion, and converging on resonant ideas. Experiential orientation was used to ensure themes reflected the overall essence of participant responses.

## Results

**Fig. 1:**
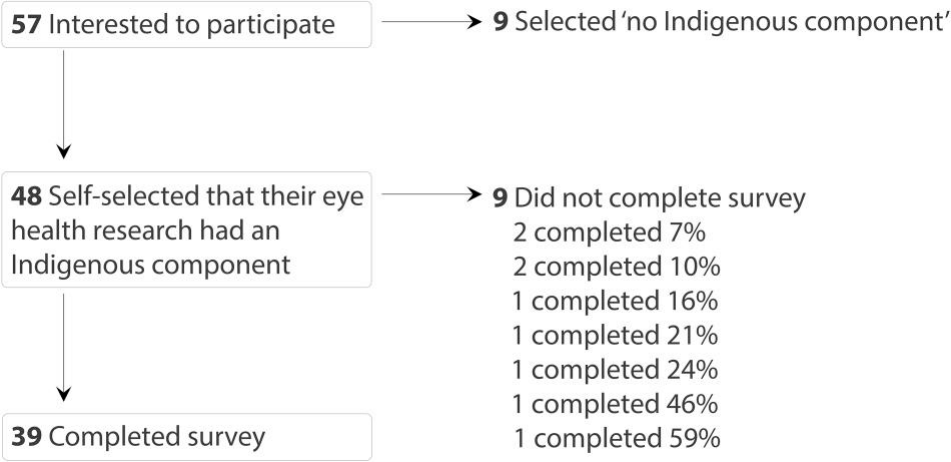
Summary of participation among people commencing the survey.

Fifty-seven individuals responded to the invitation to participate. Of these, 48 thought their eye health research included an Indigenous component (Box 1) and proceeded to the item-specific questions, with 39 of these completing all questions (39/48 = 81%; Fig. 1). No duplicate responses were identified. Data are presented from the 39 participants completing all questions.

Participants were from nine countries, with the largest group primarily conducting their research in Aotearoa New Zealand where the study was initiated (*n* = 11/39, 28%; Table 1); two participants (5%) identified as Indigenous. Almost two-thirds of participants (*n* = 24, 62%) selected more than one type of eye health research (mean of 1.8, SE+/−0.9), with epidemiology (*n* = 24, 62%) and health systems research (*n* = 20, 51%) most commonly selected (Table 1). Just over one-third of participants had heard of the CONSIDER statement before participating in this survey (*n* = 15/39, 38%).

**Table 1:** Summary of characteristics of 39 participants who conduct eye health with an Indigenous component.

Participant characteristic	*n*	%
Country		
Aotearoa New Zealand	11	28
Australia	10	26
Colombia	6	15
Brazil	5	13
Canada	3	8
Argentina	1	3
Guatemala	1	3
Panama	1	3
Peru	1	3
Type of research (72 responses)*		
Epidemiology	24	62
Health systems	20	51
Clinical	17	44
Vision science	5	13
Health education	4	10
Other (anthropology, socio-cultural research)	2	6
Indigenous component of research (114 responses)*		
Impacts the health of Indigenous peoples	36	92
Takes place on Indigenous land	26	67
Identity or membership of an Indigenous community is used as a variable for data analysis	21	54
Identity or membership of an Indigenous community is used as a variable for participation	19	49
Seeks Indigenous knowledge	12	31

^*^Could choose more than one option so adds up to >39

Participants tended to identify that their research had more than one Indigenous component (mean 2.9, S.E+/−1.5). The components most selected by participants were ‘research which impacts the health of Indigenous peoples’ (*n* = 36, 92%) and ‘research that takes place on Indigenous land’ (n = 26, 67%, Table 1).

**Fig. 2:**
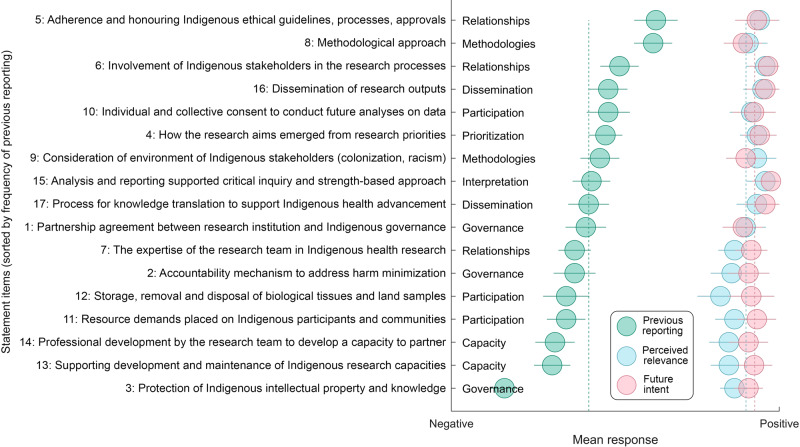
Perspectives on each CONSIDER item of 39 eye health researchers who have conducted research with an Indigenous component. Green dots represent mean results for frequency of previous report (negative=never, positive=always), blue dots represent mean results for perceived relevance for eye health (negative = ‘not important, positive=very important), pink dots represent mean results for future intent to report (negative=very unlikely, positive=very likely). Error bars are standard error of the mean. Statement items are sorted by frequency of previous reporting (green).

Across participants and items, mean reporting of CONSIDER items in previous work (from 1 = never to 4 = always) was 2.26 (+/−1.14), indicating that on average, reporting occurred ‘sometimes’ (Fig. 2, blue dotted line). Previous reporting varied by item, while the perceived relevance and intent to report in the future were high for all items (Fig. 2).

Only three items (5, 6 and 8) were previously reported ‘most of the time’ (mean score >2.5). Items 5 and 6 are within the ‘Relationship’ domain. Item 5 is articulated as ‘*Specify measures that adhere and honour Indigenous ethical guidelines, processes, and approvals for all relevant Indigenous stakeholders, recognizing that multiple Indigenous partners may be involved*’ (score = 2.87) and item 6 is ‘*Report how Indigenous stakeholders were involved in the research processes*’ (score = 2.54). Item 8 is within the ‘Methodology’ domain, articulated as ‘*Describe the methodological approach of the research including a rationale of methods used and implication for Indigenous stakeholders*’ (score = 2.85). By domain, ‘Relationship’ and ‘Methodology’ were most often reported previously (Relationship: 2.51 ‘most of the time’ and Methodology: 2.61, ‘most of the time’).

Only one item (3) was previously reported ‘never’ (mean score <1.5). Item 3 was *‘specify how the research partnership agreement includes protection of Indigenous intellectual property and knowledge arising from the research, including financial and intellectual benefits generated*’ (score = 1.49). With items pooled by domain, ‘Capacity’ was least reported by participants in past research (average score = 1.93 ‘sometimes’). This included Item 13 ‘*Explain how the research supported the development and maintenance of Indigenous research capacity*’ and Item 14 ‘*Discuss how the research team undertook professional development opportunities to develop the capacity to partner with Indigenous stakeholders’*.

The few responses given by the nine participants with unfinished surveys suggested a less optimistic perspective than that captured within completed surveys. Incomplete surveys tended to report less awareness of the CONSIDER statement, higher previous reporting of items, less perceived relevance, and less intent to report in the future, compared to responses given by those who completed the survey.

### Qualitative data

From the qualitative data we generated three themes: (1) Overlap with other guidelines, (2) Challenges with implementation (3) Integration of domains leads to cohesive, genuine and effective research (Table 2).

**Table 2:** Summary of themes with examples of associated participant quotes.

Overlap with ethical requirements	Challenges with implementation	Perspective shift to genuine and effective research
• “In the interests of concise reporting, I think this can be assumed as part of Ethics approval.” • “Again, I think this is something covered in Ethics approval and not needed in the manuscript” • “I have reported the descriptions of how Indigenous stakeholders were involved in the research processes in ethics application, but not in the peer-reviewed report yet!” • “I believe that the strategies used to encourage the participation of Indigenous people in studies that benefit them should be described in the study or research protocols and reviewed by the ethics committee, but I do not think it is necessary to report it in the final report or publication” • “I’m not sure that formal Indigenous ethics exist beyond the general ethics. I’m not sure I’d know what formal pathways to do down to ensure I was getting all the appropriate approvals. If there are ones I don’t know about, I would like to know about them.”	**Knowledge gaps:** • “This seems important, but like ‘governance’ it is unclear to me how to do this in future.” • “I’m not totally sure what these are or how to find out about them.” • “Unsure what professional development opportunities would be available.” **Does it apply to me?:** • “it is not relevant to my research” • “Due to the nature of the research carried out with the Indigenous population, it has never been an objective to identify opportunities to strengthen the capacity of association with the Indigenous parties” • “A large proportion of studies on eye health in Indigenous communities are observational epidemiological studies that do not generate intellectual property or have direct relevance to commercial or discovery science projects, and this item may have very little relevance.” • “I’m not sure I do the type of research that would lend itself to future analysis. Adding in the consent for it, when I can’t see it happening seems maybe unnecessary?” • “Might depend on the type of research / the scale of Indigenous involvement (e.g. Māori participants within large study vs Māori focus)” **Is the bar too high?** (research structure might need to change – allowing time/resource for this) • “Clinical research requires ample paperwork before it can be commenced. Adding another layer is likely to discourage already over-burdened researchers, especially early career without significant financial support or mentorship” • “Although having a formal agreement is ideal, it can be a long bureaucratic process that can cause delays in the implementation of the research projects.” • “There is a lot of work that needs to be done to shift practice in this area - it requires both cultural governance and advocacy from allies to occur. From experience this requires specific knowledge and skills e.g. in law and intellectual property to ensure protection of Indigenous knowledges and benefit for Indigenous peoples and places. Research teams and projects don’t necessarily have budget to for these tasks either, despite their importance” **Research structure, review and publication might need to change:** • “Western lens is often described by degrees etc which is often communicated in a paper however, especially with qualitative research, a standpoint is much broader, this later can be challenging when you are working with word constraints.” • “The finer detail of this information will be available upon request or as supplementary material, depending on the journal or means of publication/dissemination.” • “sometimes it is difficult to get this across in peer-reviewed journals where the editors don’t necessarily understand” • “given the word limits imposed on authors by journals, it may be difficult to prioritise including the details of this process of dissemination of findings to the relevant Indigenous stakeholders in a paper, as this may be at the expense of some other details that are more relevant to the research itself” What might help (among participants saying they were unlikely to report an item in future)*: • “More information about these agreements” • “Organisation support” / “Recommendations on what the research team can undertake in terms of PD opportunities” • “Understanding when this should and shouldn’t be included” / “An example of how this could be included in a way that made sense in the reporting could help me see how this could fit in” • “If the relevant guidelines/principles are included within CONSIDER, and updated annually, perhaps that would work best as a checklist.”	• “Aboriginal Community Controlled Health Organisations (ACCHOs) were not always representative of the needs, perspectives, priorities of the peoples and places we were working with. Since then, my work has taken a different approach to governance that is centred on cultural authority e.g. cultural governance - which ensures that traditional cultural knowledge holders in the place where the research is being conducted are the ones who govern the research and approve key decisions on all aspects of the study” • “Because all my work is led or in collaboration with Indigenous leaders, the research analysis and interpretation are strength-based and inclusive of Indigenous values” • “We collaborate with the community, so eventually they will have ownership of the data to create eye care programs that are unique to their communities, preventing blindness and encouraging eye health” • “I undertook cultural awareness training, but my real learning came through building relationships with cultural knowledge holders” • “We still have examples of neo-colonial capacity building projects - how can these be avoided?” • “I always frame this as reciprocal -e.g.knowledge exchange because it is always reciprocal!” • “We have a high level of expertise that allows us to establish good relations with the community and positively affect the territories.” • “Feedback to the community is integral to our work, and it is how we have been able to translate research findings relatively quickly. For example, after research found that there were high rates of depression and suicide in a community, the research team set a chain of events that increased the necessary support services.” • “Community meetings, “mingas”, fairs and collective meetings were held to socialize the results of the research. These were attended by Indigenous people and decision makers. Likewise, community information dissemination strategies were generated, using local indigenous radio stations” • “To support the right to self-determination, the Indigenous communities involved in the research should advise how they wish for results to be communicated and shared and they should be supported to implement the findings in a way that is most relevant to their cultural, spiritual, psychological, environmental and physical needs.” • “Once the results are obtained, a meeting is convened with the community so that they know the analysis done and so that they can contribute to it, and these comments are integrated into the final version that is delivered to the health authority and with which a socialization is made.” • “In work I have led all dissemination was collaborative and included Aboriginal community based researchers as authors who took the lead in specific and place-based report backs to Aboriginal partner organisations.”

#### Overlap with other guidelines

Participants highlighted perceived overlap between the CONSIDER statement and existing guidelines for conduct and/or reporting of research. This included institutional (e.g. university or political) and local Indigenous ethical approval pathways (e.g. OCAP [Ownership, control, access and possession] [17], ACCHO [Aboriginal Community Controlled Health Organisations]), and some discipline specific methodological guidelines (e.g. some qualitative approaches). Participants questioned whether CONSIDER needed to be reported overtly when adherence to other guidelines overlapped with the content of an item of the CONSIDER statement, highlighting some confusion between research conduct and research reporting. For example, although participants had tended to report some CONSIDER items quite often (including items 5, 6 and 8), they commonly explained that this was done for reasons other than use of the CONSIDER statement.

#### Challenges with implementation

Despite seeing the value of the CONSIDER items, many participants acknowledged the difficulty of implementing them. Participants appeared to have difficulty if connections with Indigenous communities, organisations or governance bodies were not perceived as present and approachable in their setting, particularly in the ‘Governance’ domain. Participants highlighted they wanted some additional guidance on when each item should be applied, and how to practically implement and report them. Beyond researcher training, the need for wider institutional support was also raised. This included the need for more time and funding from the employing institutions (in particular for small groups or emerging researchers), as well as increased understanding of the CONSIDER statement by journals, including reviewers and editors. Insufficient networks, knowledge and institutional support were highlighted as barriers for eye health researchers to engage in future reporting, despite good intentions.

#### Perspective shift towards genuine and effective research

Several participants appeared to be well-informed on the topic and work within institutions that support genuinely collaborative research. Participants described examples where perspective shifts had occurred for eye health researchers away from assumed Western superiority, toward a genuine appreciation and incorporation of Indigenous perspectives. This included a growing acknowledgement of Indigenous communities as holders of invaluable knowledge relevant for effective research; and view that genuine collaboration (including self-reflection and integration of items) is the only way eye health research will contribute to improved wellbeing and equitable outcomes for Indigenous peoples. However, there was concern in the group that formalising this process could diminish its impact by reducing interactions to a tokenistic approach. One example is within the ‘Capacity’ domain, where being explicit about ongoing mentorship with an Indigenous researcher may trivialise the relationship.

## Discussion

We assembled a group of 39 researchers from nine countries who have conducted and reported eye health research with an Indigenous component. Just over one-third of participants had heard of the statement prior to the survey, and there was some confusion about potential overlap between CONSIDER and other guidelines for ethical research with an Indigenous component. On average, participants disclosed only ‘sometimes’ reporting items outlined in the CONSIDER statement in the past, and the items most often reported were those that seemed to overlap with other guidelines. On the whole, participants indicated the items are relevant, and that they intend to report them in the future.

The responses regarding perceived relevance and intended future use of the CONSIDER statement are encouraging, given its potential to contribute to health equity for Indigenous populations [13, 25]. Examples of its potential have been described by Australian researchers, and included encouragement to research teams to conduct culturally respectful research with clear partnership and shared goals between Indigenous and non-Indigenous parties [26], and to benefit both researchers and the Indigenous communities in which the research was conducted [27].

Participants suggested enabling processes to enhance the uptake and impact of CONSIDER, including suggestions to foster genuine (not tokenistic) engagement, to facilitate regular updates by a broad team of people to reflect values of Indigenous groups globally, and to promote uptake by relevant institutions (including funding, employment and publishing bodies). In terms of researcher level engagement, participants felt that further training and support is needed about when and how to report CONSIDER items, especially for emerging researchers or groups.

We identified a possible knowledge gap among participants on Indigenous data sovereignty, given that CONSIDER item 3 ‘*protection of Indigenous intellectual property and knowledge’* was least often reported by participants. Indigenous data sovereignty acknowledges that data ownership and usage is subject to the nation or community from where data are gathered, rather than subject to laws of the nation or community in which data are stored [28]. Data sovereignty is important for Indigenous communities because historical engagement between Indigenous peoples and the collectors of data have often been discriminatory, with data being used as an instrument of colonisation [17, 29]. Researchers who recently applied the CONSIDER statement in participatory research in Australia recommended that it should include greater provisions to protect intellectual property and data through a prospective and explicit formal research agreement [27, 30]. Our results support this by highlighting that eye health researchers want more information about the wider ‘Governance’ domain so that it can be more easily applied in future research. This could be achieved by strengthening local networks and providing more clarity in global guidelines, and may include training, mentorship, workshops, coaching or internships.

Our survey revealed that items within CONSIDER’s ‘Capacity’ domain were infrequently reported. Although there are several contributing factors, the participants reported a reluctance to inadvertently tokenise valued relationships with their Indigenous collaborators. A fear of tokenism has also been observed in other research fields, including by those involved in Indigenous health policy development where historically, explicit Indigenous policies have been used to enforce cultural assimilation [31]. Our participants thoughtfully signalled the need to ensure the implementation of the CONSIDER statement does not devolve into a ‘box ticking’ exercise, a consideration for future research and debate. The intent of Indigenous knowledge exchange is specifically to combat these fears through promoting Indigenous leadership.

Participant responses highlight the importance of publishing and reporting standards to encourage equitable eye health research. In their comments, some participants described how research *conduct* aligned with CONSIDER items, but they questioned whether *reporting* it was necessary. There are a range of local guidelines researchers can draw on to inform research conduct, including OCAP, NACCHO [National Aboriginal Community Controlled Health Organisation] [17, 32], the Aboriginal and Torres Strait Islander quality appraisal tool [33] and institutional ethics. Reporting serves a specific function in promoting transparency, and increasing the reliability and value of published research (as outlined by the EQUATOR network, equator.org) [34]. Currently, CONSIDER is the only reporting guideline for research with an Indigenous component included in the EQUATOR network, an affiliation which can influence journal policy. Although journals endorsing a reporting guideline does not necessarily improve reporting, requiring adherence to a guideline appears to [35]. Therefore, eye health journals have an important role to play in improving the reporting of eye health research with an Indigenous component by requiring authors to use CONSIDER [36].

Our findings must be considered in the context of several limitations. Primarily, our sample is likely biased towards people who are motivated to incorporate the CONSIDER statement into their future work, as we purposely sought participants who were active in research with an Indigenous component. All researchers active in colonised countries in which Indigenous people did not cede sovereignty could be considered to meet the criteria for doing research with an Indigenous component because they conduct research on what is arguably unceded Indigenous land (Criterion 5 in Box 1) [37–40]. Furthermore, eye health research findings in colonised countries will have implications for the health of Indigenous peoples (Box 1). However, ~1 in 6 people who commenced the survey could not identify an Indigenous component to their work so were considered ineligible to progress. Further, almost 1 in 5 eligible participants who commenced the survey did not complete it and the responses collected from these individuals painted a more pessimistic picture than the responses that we have reported here. Together, our results likely overestimate the support for the CONSIDER statement within eye health researchers more broadly. However, we believe the participant group—most of whom are listed in the study author group—represent a core group of people who can work together to create change in the field. A further limitation of our study was that we were unable to achieve broader engagement—more than half of our participants were from either Australia or Aotearoa New Zealand and we recruited very few people from North America and none from Asia. It is unclear whether participants from under-represented regions may have responded differently to the results presented here. The lower participation from some global regions may reflect weaker networks of our research team in those regions, as well as less interest in and/or comfort with the project among eye health researchers in these under-represented regions. We hope that through the Indigenous Peoples Special Interest Group of the International Agency for the Prevention of Blindness that the profile of Indigenous eye health can grow, and future activities will engage researchers active in more countries.

Applying the CONSIDER statement within this current paper provided an opportunity for self-reflection within our own research. We debated which items applied to this study and how to report them (supplementary table 3). In comparison to the procedural process of reporting items for the CHERRIES checklist (supplementary table 2), we found reporting against the CONSIDER guidelines to be more challenging. Our three main reflections were the need for non-Indigenous researchers to demonstrate humility, for Indigenous researchers to show courage and boundary-setting, and all fostering safe spaces for honest and nuanced discussion about sensitive issues [13]. However, as a team of Indigenous and non-Indigenous eye health researchers, we were determined to find a way forward and realise our aspirations for improving the eye health landscape and realising equity [5, 13, 41, 42]. We encourage other eye health researchers to do the same.

CONSIDER and similar tools can help researchers embark on a new wave of Indigenous eye health research. We hope that this report will increase awareness and use of the CONSIDER statement within the eye health research community. Critical research and continued debate are needed to ensure the CONSIDER statement remains up to date to cover issues relevant for the varied Indigenous communities across a wide range of countries and legislative arrangements [12]. We are encouraged by Indigenous research methods and guidelines already in use, including Kaupapa Māori research in Aotearoa New Zealand [43]. Further, we endorse the view that rather than the majority of research being focused on quantifying the extent of health inequality experienced by Indigenous peoples, there is a need to shift towards solution-focussed research [44]. We call for other eye health researchers to join us in our commitment to use and advocate for the CONSIDER statement to facilitate good quality research that promotes equity in eye health for Indigenous peoples globally.

## Summary

### What was known before:

- Indigenous Peoples have worse access to and outcomes from eye health services and subsequently have poorer eye health compared to non-Indigenous populations.
- The CONSIDER statement (CONSolIDated critERia for strengthening the reporting of health research involving Indigenous peoples) was developed by Indigenous researchers for use by all researchers to enhance the design and reporting of their research with an Indigenous component, such as by honouring Indigenous principles and engaging Indigenous communities, governing bodies and researchers.

### What this study adds:

- We established an Indigenous Eye Health Research Consortium of 39 eye health researchers from nine countries who have reported research with an Indigenous component; these researchers had generally only ‘sometimes’ reported CONSIDER items previously, but saw value in their use in future reporting.
- We discuss the challenges and opportunities for increasing use of CONSIDER, and call for other eye health researchers to join us in our commitment to use and advocate for the CONSIDER statement to facilitate good quality research that promotes equity in eye health for Indigenous peoples globally.

### Supplementary information

Supplementary Annex

Collected data on CONSIDER items

## Data Availability

Collected responses are available as supplementary information.
